# Long-Term Ovarian Function Assessment After Haematopoietic Stem Cell Transplantation in Female Sickle Cell Anaemia Survivors

**DOI:** 10.7759/cureus.58195

**Published:** 2024-04-13

**Authors:** Olusola Olowoselu, Kehinde S Okunade, Olufemi A Oyedeji, Nosimot O Davies, Obiefuna I Ajie, Ademola Adewoyin, Gaurav Kharya

**Affiliations:** 1 Haematology and Blood Transfusion, College of Medicine, University of Lagos, Lagos, NGA; 2 Obstetrics and Gynaecology, Lagos University Teaching Hospital, Lagos, NGA; 3 Obstetrics and Gynaecology, College of Medicine, University of Lagos, Lagos, NGA; 4 Chemical Pathology, College of Medicine, University of Lagos, Lagos, NGA; 5 Haematology, Oncology, and Bone Marrow Transplant (BMT) Unit, Apollo Hospitals, New Delhi, IND

**Keywords:** fertility preservation, sickle cell disease, follicle-stimulating hormone, bone marrow transplantation, anti-müllerian hormone

## Abstract

Background: Haematopoietic stem cell transplantation (HSCT) is a potentially curative treatment for sickle cell anaemia (SCA). While HSCT offers the possibility of disease remission, it can also lead to long-term complications, including gonadal dysfunction and premature menopause.

Methods: We conducted a retrospective cohort study of female survivors who had hydroxyurea therapy and those who underwent post-HSCT follow-up for SCA at a teaching hospital in Lagos, Nigeria, between January 2019 and December 2022. Participants were eligible if they were at least five years post-HSCT or hydroxyurea treatment and had available serum samples for markers of ovarian function measurement. Demographic and clinical data were collected from the hospital register and patients’ medical records. Serum levels of oestradiol, luteinizing hormone (LH), follicle-stimulating hormone (FSH), and anti-Müllerian hormone (AMH) were measured using the Abbott Architect i1000SR chemiluminescent immunoassay analyzer (Abbott Diagnostics, Abbott Park, IL). Descriptive statistics and inferential analyses were used to assess the relationship between markers of ovarian function (FSH and AMH) and clinical parameters.

Results: There were statistically significant differences in the median serum levels of all the assessed endocrine hormones between the HSCT and non-HSCT (hydroxyurea) groups of SCA survivors. Up to 82.6% of the SCA survivors experienced ovarian dysfunction after HSCT treatment. Impaired ovarian function in SCA survivors was associated with a longer median follow-up duration than in SCA survivors who had normal ovarian function (12.0 vs. 7.5 years, p = 0.048). There were higher odds of impaired ovarian function in the SCA survivors who had myeloablative regimens than in those who had reduced intensity conditioning regimens (94.1% vs. 50.0%, p = 0.040).

Conclusion: Our study highlights the significant impact of HSCT on long-term ovarian function in female SCA survivors. However, further prospective studies with larger sample sizes and longer follow-up periods are required to confirm our findings and elucidate the factors influencing ovarian function in SCA survivors of HSCT. In addition, studies are also needed to further elucidate the optimal transplant protocols and fertility preservation strategies to minimize gonadal toxicity and preserve reproductive potential in female SCA patients undergoing HSCT.

## Introduction

Sickle cell anaemia (SCA) is a hereditary blood disorder characterised by abnormal haemoglobin (Hb) molecules in red blood cells, leading to various complications, including anaemia, pain crises, and organ damage [1]. For decades, haematopoietic stem cell transplantation (HSCT) has emerged as a potentially curative treatment option for SCA, offering hope for improved quality of life and longevity for affected individuals [2]. Haematopoietic stem cell transplantation involves the infusion of healthy stem cells into the patient's bloodstream, which can then repopulate the bone marrow and produce normal red blood cells [3]. However, HSCT can also lead to long-term complications, including gonadal dysfunction and premature menopause, which are significant concerns for female SCA survivors [3-5].

While HSCT holds promise as a therapeutic intervention for SCA, its long-term impact on ovarian function and fertility in female survivors has not been adequately studied, especially in developing countries, including Nigeria, where SCA prevalence is highest [6]. The importance of assessing ovarian function following HSCT in female SCA survivors cannot be overstated. This is because the preservation of ovarian function and fertility is crucial for these individuals as they navigate the challenges of living with a chronic illness [5]. It will also ultimately inform clinical decision-making, guide reproductive counselling and the development of supportive interventions, and drive future research initiatives aimed at optimising the care and well-being of female SCA survivors in our community and beyond.

Against this backdrop, our current retrospective cohort study investigated long-term ovarian function through a comprehensive assessment of markers of ovarian function, including serum oestradiol, luteinizing hormone (LH), follicle-stimulating hormone (FSH), and anti-Müllerian hormone (AMH) levels in SCA survivors who underwent HSCT and their comparative counterparts treated with hydroxyurea (non-HSCT) in Lagos, Nigeria. We also compared the ovarian function between myeloablative and reduced intensity conditioning approaches in SCA survivors who underwent HSCT treatment. By shedding light on the long-term effects of HSCT on ovarian function in female SCA survivors, our study will contribute to the body of evidence surrounding the reproductive health and quality of life of individuals with SCA who undergo HSCT.

## Materials and methods

Study design and settings

This retrospective cohort study was conducted among Nigerian female SCA survivors who underwent HSCT at the bone marrow transplant centres of the Institute de Mediterranean in Rome, Italy, and Indraprastha Apollo Hospital in New Delhi, India, after referral, and their comparative counterparts who had hydroxyurea therapy at the Lagos University Teaching Hospital (LUTH) in Lagos, Nigeria, between January 2019 and December 2022. The LUTH is the teaching hospital of the College of Medicine, University of Lagos. It is a referral centre for private and public hospitals in Lagos State and its surrounding states of Ogun and Oyo. It has a comprehensive haematology and blood transfusion department that provides primary care to SCA patients and post-HSCT follow-up care to SCA survivors after their initial referral to and completion of HSCT treatment at the partner/collaborating institutions at the Institute de Mediterranean and Indraprastha Apollo Hospital. Patients with SCA referred for HSCT in these two institutions underwent similar treatment using the same protocol and guidelines.

Study population

Eligible study participants were female SCA patients who had undergone HSCT or had used hydroxyurea (non-HSCT) for SCA treatment with subsequent routine hormonal testing (for ovarian function assessment) as part of their follow-up monitoring at least five years after treatment initiation. The inclusion criteria included female SCA survivors who had available serum sample measurements of oestradiol, LH, FSH, and AMH at least five years following their treatment. Excluded from the study were patients who died or were lost to follow-up post-HSCT or non-HSCT (hydroxyurea) treatment.

Study procedure and data collection

Demographic and clinical data, including the patient’s age at treatment, type of treatment (HSCT or non-HSCT), donor type (matched sibling or haploidentical), and conditioning regimen (myeloablative or reduced intensity), were collected from the hospital's medical records. Other relevant information collected after follow-up included the results of participants’ post-treatment Hb and hormonal status, including serum oestradiol, LH, FSH, and AMH levels, menstrual history, and duration since transplantation. Serum levels of oestradiol (in pg/mL), LH (in mIU/mL), FSH (in mIU/mL), and AMH (in ng/mL) were measured using the Abbott Architect i1000SR chemiluminescent immunoassay analyzer (Abbott Laboratories, Abbott Park, IL) according to the manufacturer's instructions. Impaired ovarian function was defined as AMH levels <1.0 ng/mL (reference: 1.0 to 3.0 ng/mL) and/or FSH levels >7 mIU/mL (reference: 0.3 to 7.0 mIU/mL).

Statistical analysis

Statistical analysis was performed using IBM SPSS Statistics for Windows version 29.0 (IBM Corp., Armonk, NY). Descriptive statistics were used to summarize participant demographic characteristics and clinical data. Categorical variables were presented as frequencies and percentages, while continuous variables were presented as means (standard deviation) or medians (interquartile range (IQR)). We handled missing data by running a missing values analysis if no pattern was detected and the multiple imputation method if a pattern was detected [7]. We tested continuous variables for normality using the Kolmogorov-Smirnov test with Lilliefors' significance correction. Median oestradiol, LH, FSH, and AMH levels at least five years post-HSCT and non-HSCT treatment were calculated, and then inferential statistics using an independent-sample t-test for normally distributed continuous variables (or Mann-Whitney U test for skewed variables) and a chi-square test (or Fisher’s exact test) for categorical variables were used to compare markers of ovarian function and other important variables such as age of SCA survivors, duration of follow-up, and Hb concentration between the two groups. Subsequently, we performed subgroup analyses comparing the median post-HSCT follow-up duration and median Hb concentration between the myeloablative conditioning regimen and the reduced-intensity regimen among SCA survivors who underwent HSCT. Statistical significance was reported at a p-value <0.05.

Ethical considerations

The research protocol for this study was approved by the research ethics committee of the LUTH (approval number: ADM/DCST/HREC/APP/3836) on November 25, 2021, according to the Declaration of Helsinki before accessing the patient’s medical records and subsequent data collection. Strict confidentiality measures were maintained for patients' information throughout and after the conclusion of the study.

## Results

A total of 23 (59.0%) female SCA survivors (HSCT group) and 16 (41.0%) comparative counterparts (non-HSCT group) were included in the study. The patients’ mean age during treatment was 9.3 (SD 3.2) years, and the median post-treatment follow-up duration was eight (IQR 6.0-12.0) years in both groups. There was a statistically significant lower mean age in SCA survivors in the HSCT group than those in the non-HSCT group (7.9 (SD 3.2) vs. 11.4 (SD 2.2) years, p<0.001).

**Figure 1:**
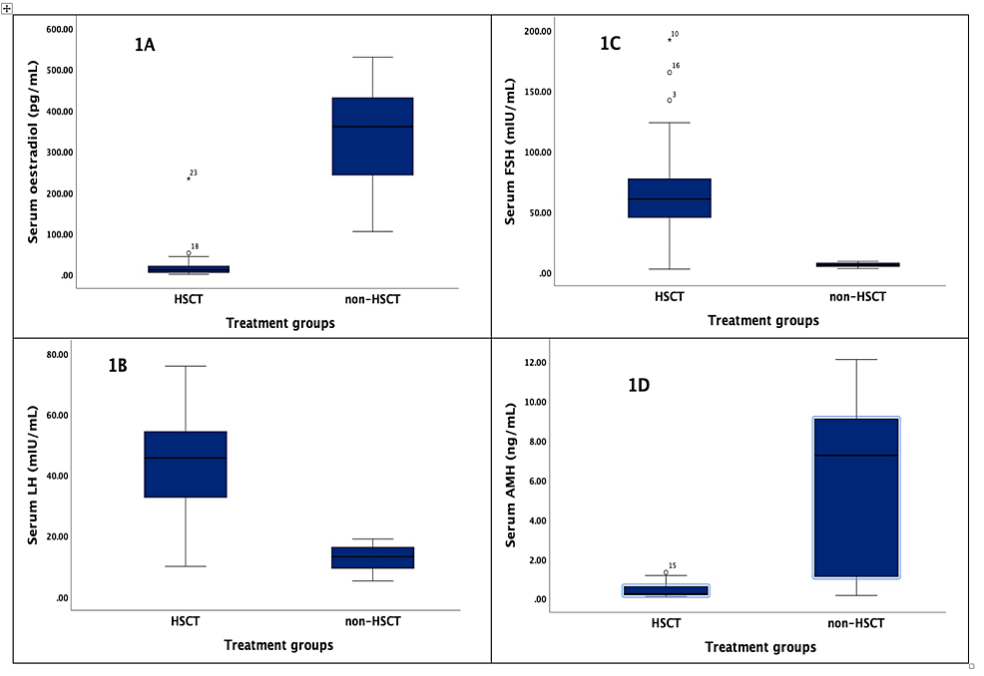
Images A-D show the comparative median serum levels of (A) oestradiol, (B) luteinizing hormone (LH), (C) follicle-stimulating hormone (FSH), and (D) anti-Müllerian hormone (AMH) in the haematopoietic stem cell transplantation (HSCT) and non-HSCT groups

The majority (82.6%) of SCA survivors in the HSCT group reported amenorrhoea, while none in the non-HSCT group reported it. The median Hb for SCA survivors in the HSCT group was significantly higher than those in the non-HSCT group (12.0 vs. 10.2 g/dL, p = 0.009). The median serum oestradiol, LH, FSH, and AMH levels were 11.9 (IQR 4.9-23.0) pg/mL, 45.6 (32.6-54.8) mIU/mL, 60.4 (IQR 45.1-80.3) mIU/mL, and 0.23 (IQR 0.18-0.70) ng/mL, respectively, in the HSCT group, and 360.5 (IQR 225.8-431.5) pg/mL, 13.2 (8.4-16.4) mIU/mL, 5.9 (IQR 4.6-7.5) mIU/mL, and 7.26 (IQR 1.11-9.30) ng/mL, respectively, in the non-HSCT group. There were statistically significant differences in the median serum levels of all the assessed endocrine hormones between the HSCT and non-HSCT groups of SCA survivors (Figures 1A-1D). We reported an ovarian dysfunction rate of 82.6% (19/23) after HSCT, compared to 18.8% (3/16) observed after non-HSCT treatment.

**Figure 2:**
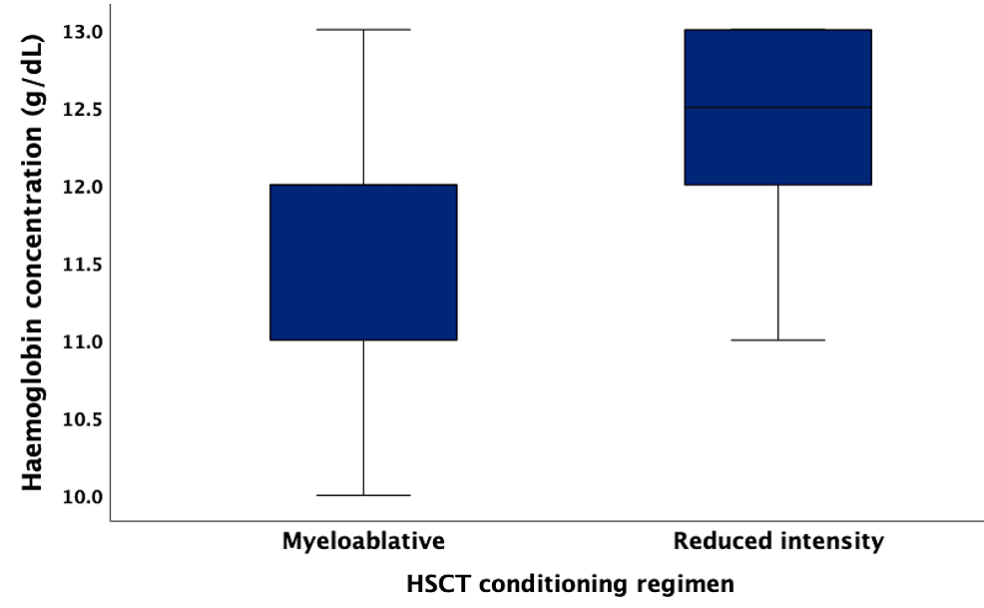
The post-haematopoietic stem cell transplantation (HSCT) median haemoglobin concentration among sickle cell anaemia (SCA) survivors was stratified by the type of HSCT conditioning regimen.

In the subgroup analyses of SCA survivors in the HSCT group, the median post-HSCT follow-up duration was 11.0 (IQR 8.0-12.0) years. There was no difference in the median Hb concentration for survivors in the myeloablative HSCT group and those in the reduced-intensity HSCT group (12.0 vs. 12.5 g/dL, p = 0.165) (Figure 2).

As shown in Table 1, 82.6% (19/23) of the SCA survivors experienced ovarian dysfunction after HSCT treatment. There was no association between the mean age of SCA patients (p = 0.110) or the median age of SCA survivors at follow-up assessment (p = 0.934) and impaired ovarian function. A longer median follow-up duration was observed in SCA survivors with impaired ovarian function than in survivors with normal ovarian function (12.0 vs. 7.5 years, p = 0.048). There were higher odds of impaired ovarian function in the SCA survivors who had matched sibling donors and myeloablative conditioning regimens compared to those who had haploidentical donors and a reduced intensity regimen (94.1% vs. 50.0%, p = 0.040).

**Table 1:** Distribution of sickle cell anaemia survivors based on ovarian function post-HSCT (n=23) HSCT: haematopoietic stem cell transplantation; IQR: interquartile range; SD: standard deviation †Fisher’s exact test; ‡Mann-Whitney U test

Factors	Ovarian function		p-value
	Impaired (%)	Normal (%)	
Participants	19 (82.6)	4 (17.4)	NA
Mean age during HSCT (±SD)	7.4 ± 2.9	10.3 ± 3.6	0.110
Median follow-up duration (IQR)	12.0 (9.0 – 12.0)	7.5 (5.5 – 12.3)	0.048^‡^
Median age of survivors (IQR)	17.0 (16.0 – 18.0)	17.0 (15.3 – 21.8)	0.934^‡^
Conditioning regimen			
Myeloablative	16 (94.1)	1 (5.9%)	0.040^†^
Reduced intensity	3 (50.0)	3 (50.0)	

## Discussion

This retrospective cohort study provides insights into long-term ovarian function in female SCA survivors after HSCT treatment in Lagos. We reported that SCA survivors had HSCT at an earlier age compared to survivors who had non-HSCT treatment. Our findings also suggest that HSCT significantly impacts ovarian function compared to non-HSCT treatment. More than four-fifths of the SCA survivors had ovarian dysfunction after HSCT treatment. Additionally, we reported that having a longer duration of follow-up post-HSCT was associated with a higher probability of impaired ovarian function. It was also noted that, despite having similar treatment effectiveness, using a reduced-intensity conditioning regimen after a haploidentical donor has less impact on ovarian function than using a myeloablative regimen with a matched donor.

It was not surprising that SCA survivors who had HSCT treatment had it at an earlier age compared to survivors who had non-HSCT treatment. This is because it is believed that performing HSCT at an earlier age in SCA patients will offer the best chance for successful engraftment, prevention of complications, and long-term benefits compared to when the procedure is performed at a relatively older age [8]. We reported that up to 82.0% of the SCA survivors in our study experienced ovarian dysfunction after HSCT treatment, similar to a previous study by Mayer et al. [9] that reported impaired gonadal function in about 80% of children who underwent bone marrow transplantation for acute lymphoblastic leukaemia in University Children's Hospital, Tübingen, Germany. Our reported figure is, however, higher than the 57.1% reported in a multicenter trial of 14 paediatric females who underwent a human leukocyte antigen (HLA)-matched HSCT for sickle cell disease in Children's Hospital and Research Centre, Oakland, California, USA, between 1991 and 2000 [10], which may be an indication of the pre-existing high prevalence of malnutrition in our setting [11], which could negatively impact the ovarian reserve of SCA patients before and after the HSCT treatment.

Similarly to most previous studies [5,12-14] that have identified gonadal dysfunction as the most common endocrine complication of HSCT in long-term female SCA survivors of HSCT, our study reported a significant impact of HSCT on ovarian function. This contrasts with the belief that young age protects the ovary from damage during HSCT [15]. This thus highlights the importance of considering the potential reproductive implications of HSCT in female SCA patients and providing comprehensive fertility counselling and preservation strategies for female SCA patients considering HSCT. Therefore, given the potential impact of HSCT on ovarian function and fertility, healthcare providers need to discuss reproductive options, including oocyte or embryo cryopreservation, with patients before transplantation. Additionally, close monitoring of ovarian function post-transplantation is warranted to identify patients at risk of premature ovarian insufficiency and to facilitate timely interventions to preserve fertility and prevent complications of premature menopause, such as osteoporosis and other complications resulting from a lack of hormones [5].

One notable observation from our study is the association between the duration of follow-up post-HSCT and the probability of impaired ovarian function. Specifically, we found that longer durations of follow-up were associated with a higher likelihood of SCA survivors experiencing impaired ovarian function. This finding suggests that the impact of HSCT on ovarian function may become more pronounced over time regardless of the age of performing HSCT, underscoring the need for long-term monitoring and early management of reproductive health in SCA survivors after undergoing HSCT. Although, in contrast to the report by Sanders in 1991 indicating that almost all female patients above the age of 12 who underwent HSCT experienced ovarian failure, possibly due to a decreased reserve of primordial follicles [4], we reported no impact of age on ovarian function in our current study. Another important finding in our study is the differential impact of conditioning regimens, despite similar treatment effectiveness, on ovarian function outcomes post-HSCT. Specifically, our results suggest that using a myeloablative regimen with a matched donor has a higher impact on ovarian function than using a reduced-intensity conditioning regimen with a haploidentical donor, which corroborates the findings from previous studies [16]. This differential impact on ovarian function by different conditioning regimens may be attributed to varying degrees of gonadotoxicity associated with different transplant protocols, despite advancements in HSCT therapeutic approaches [3]. Reduced-intensity conditioning regimens, associated with lower doses of chemotherapy and radiation, may result in less damage to ovarian tissue and preservation of ovarian function compared to myeloablative regimens [3,16]. Therefore, our findings suggest further research to elucidate the mechanisms underlying gonadal toxicity in HSCT and to optimize conditioning regimens to minimize adverse effects on ovarian function. It also underscores the importance of regimen selection in mitigating the adverse effects of HSCT on ovarian function, with potential implications for fertility preservation and reproductive health counselling in female SCA patients undergoing HSCT.

The major strength of this pilot study is the longitudinal data collection approach, which allows for the assessment of temporal trends and provides a more nuanced understanding of the impact of HSCT on ovarian function. Furthermore, this is the first study to generate data on the long-term ovarian function patterns of Nigerian SCA children who underwent HSCT treatment. However, despite the valuable insights provided in this study, several limitations warrant consideration. The study's retrospective design could introduce bias and limit the generalizability of our findings. Additionally, the sample size may have been insufficient to detect subtle differences in ovarian function outcomes between treatment groups. Therefore, future prospective studies with larger cohorts and longer follow-up periods are warranted to validate our findings and further elucidate the factors influencing ovarian function post-HSCT in female SCA survivors.

## Conclusions

Our study highlights the significant impact of HSCT on long-term ovarian function in female SCA survivors. This underscores the need for tailored reproductive health management strategies, including fertility counselling, preservation strategies, and hormone replacement therapy. However, further prospective studies with larger sample sizes and longer follow-up periods are warranted to confirm our findings and elucidate the factors influencing ovarian function in SCA survivors of HSCT. In addition, studies are also needed to highlight the optimal transplant protocols and fertility preservation strategies to minimize gonadal toxicity and preserve reproductive function in female SCA patients undergoing HSCT.
